# Pretesting a Poster on Recommended Stress Management During the COVID-19 Pandemic in Indonesia: Qualitative Study

**DOI:** 10.2196/25615

**Published:** 2021-09-23

**Authors:** Risa Laras Wati, Annisa Sayyidatul Ulfa, Zulfa Kevaladandra, Shelly Shalihat, Bella Syahadatina, Hadi Pratomo

**Affiliations:** 1 Department of Health Education & Behavioral Sciences, Faculty of Public Health Universitas Indonesia Depok Indonesia

**Keywords:** pretesting, media, stress, COVID-19

## Abstract

**Background:**

The COVID-19 Peritraumatic Distress Index (CPDI) is a self-report questionnaire developed to evaluate the frequency of anxiety and depression symptoms among individuals during the COVID-19 pandemic. A recent study in China showed high CPDI scores among individuals in the 18-30 years age group and those over 60 years. During the COVID-19 outbreak, people were expected to maintain their mental health conditions, especially stress levels. Therefore, many national governments actively published health promotion media in an effort to educate the public. One such media developed by the Ministry of Health, Republic of Indonesia, was a poster titled “Hindari Stres dan Tetap Optimis dengan Melakukan Aktivitas Sehari-hari dan Tetap Menjaga Jarak.”

**Objective:**

The aim of this study is to conduct a test on a stress management recommendation poster developed by the Ministry of Health, Republic of Indonesia, in response to the COVID-19 outbreak by using pretesting communication theory.

**Methods:**

In-depth interviews were conducted among 8 key informants and 1 graphic design expert.

**Results:**

Pretesting can identify the strengths and weaknesses of media. The large amount of text and the lack of illustrations made the poster less attractive to readers. Moreover, there was a discrepancy between the title and contents of the poster. The poster was not able to persuade the informants to change their behavior in the near future.

**Conclusions:**

The poster was understood and accepted by the informants, but there was still much to be improved considering the poster was a product of the Ministry of Health, Republic of Indonesia.

## Introduction

In December 2019, a new respiratory disease manifesting as viral pneumonia emerged in Wuhan, China [1]. COVID-19 is caused by the novel coronavirus and is known to spread from person to person and is prevalent among young to older adults, affecting individuals of the age group 30 to 79 years [2,3]. In the first week of March 2020, the first COVID-19 case was confirmed in Indonesia, and the emergence of fear had a positive impact on the citizens with regard to the demonstration of the preventive behavior of purchasing personal protective equipment [4].

Many people were stressed and depressed because the emergence and patterns of COVID-19 transmission were unclear [5]. The COVID-19 pandemic has posed a serious threat to the society and triggered psychological challenges such as stress, anxiety, and depression [6]. *Stress* is a form of perceived threat with *anxiety* causing discomfort, emotional tension, and difficulty adjusting [7].

A study in China used the COVID-19 Peritraumatic Distress Index (CPDI) to determine the frequency of anxiety and depression symptoms among people. The results of this study showed that individuals aged between 18 and 30 years and those aged above 60 years showed high CPDI scores. The high scores reported among young adults (18-30 years) seem to confirm the findings from previous studies that young adults tend to receive a large amount of information from social media, which can easily trigger stress [6]. A CPDI-related study conducted in Indonesia showed that 36.5% of the respondents had mild to severe distress. Among this proportion of respondents that experienced distress during the COVID-19 pandemic, the majority was <30 years old (39.5%) and female (37.9%) [8]. The highest level of stress was reported by those at work, that is, people who were concerned about their exposure to the virus while using public transportation to commute to work and delays in work time. Moreover, an anticipated drop in income could further explain the high stress levels reported among individuals [6].

During the COVID-19 pandemic, it is essential that people maintained a positive mental condition. Some of the ways to maintain a healthy mental state include positive thinking, doing things that bring out positive emotions (such as entertaining activities at home, hanging out with family), and engaging in sports. In addition, spirituality also plays an important role in maintaining one’s mental well-being [9]. The Indonesian government had actively developed policies, formed a COVID-19 task force, provided directions, and published health promotion media to educate the public [10]. Until now, there has been no research that discussed the trial of poster media issued by the Ministry of Health, as per the statement by the head of the sub-directorate for Information, Education and Communication (IEC) of the directorate of Health Promotion and Community Empowerment, Ministry of Health, Republic of Indonesia. The health promotion sector had not conducted a trial to evaluate the readability of the guidebook prepared for COVID-19 prevention in the community. Therefore, we intended to evaluate the readability of the abovementioned guidebook from the public’s perspective. Improvements highlighted and existing input could be used for poster development in the future.

This study aims to understand the public’s comprehension of health promotion media in the form of a poster through five variables, including attention, comprehension, acceptability, self-involvement, and persuasion [11]. These five variables can illustrate the comprehension of the public on health promotion media issued by the Ministry of Health, Republic of Indonesia, titled “Avoid Stress and Stay Optimistic by Doing Daily Activities and Keeping Distance,” which is the recommended media for stress management [10]. The results of this study are expected to provide insights into the use of the media by pretesting communication for the community and related stakeholders.

## Methods

### Study Design

This study used a qualitative evaluation method of quality control pretesting. In 2015, Windsor stated that this method aims to document the target audience’s perception of messages conveyed through written, visual, and audio media. This pretesting was also an important early step to ensure the quality and competence of data of the media [12].

### Informants

The key informants in this study were 8 women aged 21 to 27 years (young adults) who lived in Jakarta, Bogor, Depok, Tangerang, and Bekasi areas (ie, Jakarta Metropolitan Area). The inclusion criteria were as follows: currently in a school-from-home (SFH) or work-from-home (WFH) condition and owns a smartphone with adequate data package. All key informants were internally coded as P1-P8. The informant criteria selection was conducted based on a similar study conducted in China that used the CPDI, which showed the stress levels of women were higher than those of men, with a mean (SD) score of 24.87 (15.03) versus 21.41 (15.97), respectively (P<.001). Moreover, the young adult age group (mean age 27.76, SD 15.69 years) was the group that reported the highest stress level due to work obligations, considering this was a productive age group [6].

### Study Instrument

**Figure 1:**
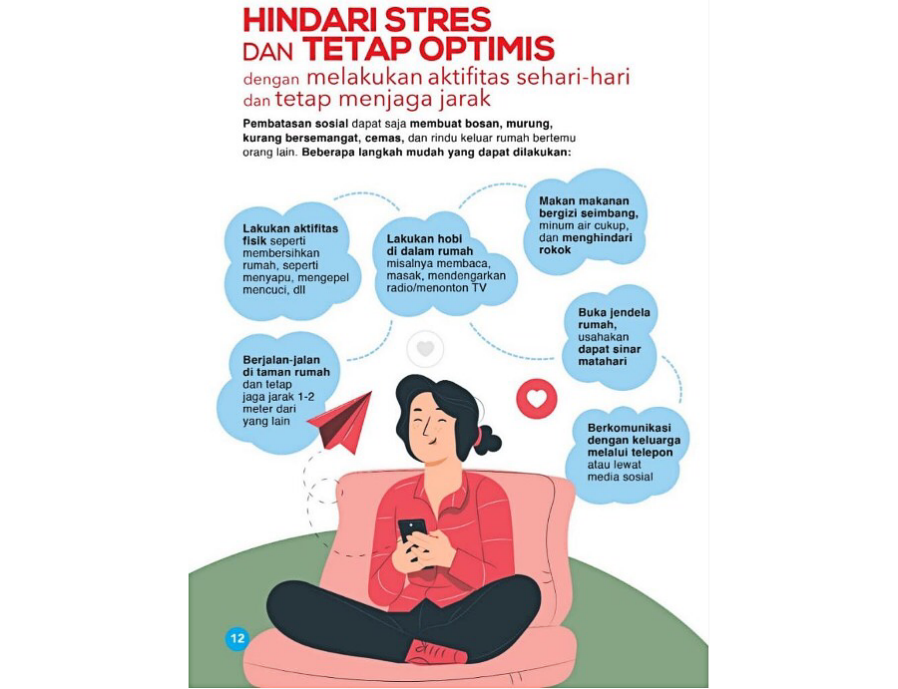
Poster evaluated in this study on the recommendations to reduce and manage stress during the COVID-19 pandemic.

We used the poster based on the guidelines issued by the Ministry of Health, Republic of Indonesia, which are published on the Health Promotion section of the Ministry of Health’s website [13] (see Figure 1). The study instrument was an in-depth interview guideline with adjusted variables based on the conceptual framework and trial information matrix. Prior to the study, the research instrument was tested on two informants similar to the target audience. The purpose of this instrument trial was to seek clarity of each question variable, the order of the questions, and interview duration, and to add the required question variables by probing what will be asked. The results of the instrument trial were then used to improve and complement the in-depth interview instrument. The duration required for an interview was 30 to 45 minutes. Before the interview, we asked the informants to look at the poster for about 10 minutes. Data collection in this study was performed based on the improved instrument.

### Data Collection

The data collection method used in this study was in-depth interviews, which were conducted from June 1 to 7, 2020. These interviews were conducted via audio calls via the WhatsApp application and by using the Zoom videoconferencing software. To increase the objectivity of the research, triangulation of sources was applied to this study, namely, by including a graphic designer. Before conducting the interview, the researcher explained the informed consent process to each informant, including the consent to use the recordings during the interview.

### Research Ethics

Medical research is subject to ethical standards that promote respect for all human beings and protect their health and rights. Some research populations are vulnerable and need special protection. Each informant was required to fill out an informed consent as required by the Declaration of Helsinki, 1975 [14]. The research objective must be ethical and protect the rights of informants in accordance with the National Guidelines for Health Research Ethics [15], including the principles of respecting human dignity (respect for person), doing good (beneficence), and not causing harm (nonmaleficence), as well as the right to justice. Issues related to research ethics were contained within the informed consent provided by the researcher before the interview with each informant commenced.

### Data Analysis

Data analysis involved completing field notes with transcripts of recordings. The transcript summary was then read out to each informant for their confirmation, thus providing an opportunity to check the authenticity of the transcript content. All informants provided their consent for data collection. Data analysis was conducted as soon as data became available; this was done so that researchers could also simultaneously plan the direction of focus on topics and discussions that were important to explore in this study [16].

## Results

### Characteristics of Informants

A total of 8 informants who lived in the Jakarta Metropolitan area interviewed along with 1 graphic designer—as a supporting informant, who was included as a triangulated source. Table 1 shows the characteristics of these study informants.

The 5 pretesting elements of the interview guide are presented in Table S1 of Multimedia Appendix 1. Information on the results was obtained based on the answers of informants using the interview guidelines. Each pretesting element had several items that were to be asked to the informants; these are described below.

**Table 1:** Characteristics of study informants.

Informant	Age (years)	Location	Activity (WFH^a^/SFH^b^)	Occupation	Source of health information	Internet use (hours/day)
Informant 1 (P1)	23	Red zone/DKI Jakarta	WFH and SFH	General employee and college student	Google, Alodokter	10
Informant 2 (P2)	23	Red zone/DKI Jakarta	WFH	General employee	Alodokter, Google	>10
Informant 3 (P3)	21	Red zone/Depok	SFH	College student	Line today, health website, Alodokter	10
Informant 4 (P4)	25	Red zone/Bekasi	WFH	General employee	Google	6
Informant 5 (P5)	21	Red zone/Bekasi	SFH	College student	Instagram and health website	10
Informant 6 (P6)	27	Red zone/Tangerang	WFH	General Employee	Health Website	10
Informant 7 (P7)	21	Red zone/Bogor District	SFH	College student	Google, electronic news media	8
Informant 8 (P8)	21	Red zone/Bogor District	WFH	General employee	Google, website MoH^c^ RI	10
Informant 9 (supporting informant)^d^	26	Bandung	WFH	Graphic designer	—^e^	—

^a^WFH: work from home.

^b^SFH: school from home.

^c^MoH: Ministry of Health, Republic of Indonesia.

^d^Employed at a private company in Bandung.

^e^Not available.

### Attention

There are several subthemes in this theme. We asked about several things such as title, design, color, font, layout, and aesthetic value. These subthemes will support the attention of the poster for audiences.

#### Title

Based on the interview with the graphic designer, the title of the poster was unattractive, and it even seemed that there was no connection between the title and the poster content. Substantially, the meaning of the word “optimistic” in the title was unclear in the context of the poster content. For example, if we look up at the phrasing “suggestion eating nutrition food,” the word “optimistic” was not found to be relevant to the sentence. Some people might actually be stressed thinking about what nutritious foods they can consume at a cheap price during the pandemic.

It would be appropriate if the word *optimistic* in the title of the poster was replaced with words that are more related to the content, such as “Avoid Stress by Staying Productive,” as the poster contains content on how to be productive while maintaining health values. The title of the poster also seemed too formal and not attractive for the readers. P5 stated that the title was suitable, but if the information on the poster was not read till the end, the poster would not show it was related to COVID-19. According to P1, the title was *very ordinary*. However, P2 had a different opinion—the title was considered persuasive, whereas P3 thought the title was representative of the contents of the poster.

#### Design

According to the graphic designer, the poster contained considerably more text than images, so it seemed *boring*. This was also in accordance with the statements of P3 and P6 who stated that the poster design was suitable but did not attract attention. This answer contradicts the opinion of P7 who stated the design was suitable.

#### Color

According to the graphic designer, the combination of colors on this poster was unsuitable; this could be seen in the writing balloons (consisting of a blue background and black text, the combination of colors may cause eye fatigue to the viewers). However, according to P1, the colors on the posters could make the reader focus on the content. P4 said the poster colors overall were suitable.

#### Font

According to several informants, the text was clear and suitable. However, according to P3 and P8, some text was too small; therefore, it was too difficult to read. P6 suggested that the text in small letters should be enlarged.

#### Layout

According to the graphic designer, this comic-like layout could make people confused from where to start reading the information. Moreover, the poster contained line elements and small plain drawings with labels and locations that were not clear. This observation was in line with the statements by P4 and P5. According to P1, it would be more suitable to add more images and reduce the amount of text in the poster.

#### Aesthetic Value

P1 rated the poster as aesthetically ordinary but still acceptable. P4 stated that the poster did not really bring out the artsy aesthetic look that is preferred by millennials. However, P8 stated that the poster was quite aesthetic enough.

### Comprehension

#### Message Content

Several informants comprehended the meaning of conveyed messages, but several others considered the message content did not match the main topic of the poster. For instance, P3 stated that the word “optimistic” was not described and created confusion; moreover, the title, images, and the message content were not related. P4 stated there were repeated messages conveyed in the poster. P5 stated that the poster content had to be read as a whole in order to understand that the poster was published in the context of pandemic prevention, especially because there were no words, sentences, or jargons related to COVID-19. P7 and P8 stated they could comprehend the message content of the poster.

The graphic designer stated that the message content of the poster seemed that no assessment was carried out referring to the results of previous studies. The contents of the existing messages were indeed basic, completed in our daily lives, and thus lacked additional insights.

#### Sentence Structure

Several informants stated that the sentence structure used in the poster content was in accordance with effective and correct writing guidelines. However, P2 reported that the message content did not relate with the main title of the poster—the main title emphasized *stress*, but the message content did not explain the causes of stress. According to the graphic designer, the sentence structure used was long winded, whereas key points should be directly mentioned after the main title.

#### Language

All informants said that the use of language or terms in the poster was already appropriate, easy to comprehend, and did not cause ambiguity. P3 suggested the language used should include more attractive diction for the millennials.

### Acceptability

#### Receiving Message Content

Results of the interview showed there were several informants who felt offended. P1 said she was not offended at all. However, P2, P3 and P7 mentioned that someone might be offended because it was practiced by the informant. However, since it was in-line with the current condition, it did not cause an issue.

#### Material Suitability Related to Norms of the Informants

Several informants stated that the material on the poster was in accordance with norms adopted by them, P2 stated that although it was in accordance with the norm, it was not enough to represent them all. According to P3, being in accordance with norms means that there was nothing contradictive with the adopted norms and that the content was insensitive.

### Self-Involvement

Several informants considered the message content was in accordance with the current situation of the informants, but according to P3 and P7 the contents of the message was not conveyed exclusively. The message content of the posters was not intended for people who work at home but for the public.

### Persuasion

#### Attractiveness Toward Persuasion

P6 stated that the posters were only in the form of warnings and was ineffective to persuade behavior as conveyed in the poster. However, P3 and P1 were of a contrasting opinion.

#### Impact of the Message Content

P4 and P6 stated that the recommendations were a reminder during quarantine, whereas P7 considered that the messages were sufficiently conveyed to the informants but were unable to cause an impact for change.

#### Plans of Informants After Reading Poster Messages

P1 and P7 stated that they will try to implement the recommendations provided by the Ministry of Health, Republic of Indonesia. P3 and P6 stated they will try to follow the suggestions conveyed via the poster other than what they have done before reading the poster. Meanwhile, P5 answered that she would try to be consistent with regard to implementing activities usually carried out in addition to the recommendations provided by the poster.

## Discussion

### Principal Findings

The poster “Hindari Stres dan Tetap Optimis” was one of the IEC media developed by the Ministry of Health concerning the guidelines titled “Panduan pencegahan penularan covid 19 untuk masyarakat.” Based on the pretesting results, the evaluation of the poster media was as follows:

#### Attention

Poster presentations are used to inform and educate participants, influence emotion, and cause behavioral change in practice. Deciding on the overall format or layout of the poster is the second important step. Because viewers are generally drawn to a poster due to its appearance, and they frequently associate the quality of research topic with the quality of the poster, it is important the poster leaves viewers with a favorable impression [2,3]. The message and all aspects of the poster should be straightforward and presented in a meaningful way [4]. It is best if the poster is able to stand on its own because the presenter may not always be present during viewing. The title of the poster should appeal to the viewer and be eye catchy. The title should not exceed 10 words or be longer than two lines. According to the basic overall results of this study, we know that the design of the poster is important especially if the poster is intended to inform and educate, influence emotion, and lead to a change in behavior of the audiences. Furthermore, the message of the poster should be straightforward and presented in a meaningful way [17]. The poster should also use a suitable typeface, layout, and aesthetic value. The results of this study suggest that a maximum of two primary colors should be used for the main text of the poster [18]. A poster that looks bright and has attractive colors can increase public interest upon viewing and reading the poster.

The title should have the largest font size to catch the audience’s attention. Keep the title short as possible [18]. Another attribute that must be considered is the technology aspect. As the graphic designer mentioned, a good poster or infographic media can be placed on any social media without reducing the quality of the design. However, this poster is appropriate only to be placed on a website. While designing, developing, testing, and implementing a message behavior change program, it is important to follow a good design process [19].

#### Comprehension

Responses of informants showed that the messages on the poster was comprehensible, supported by the use of appropriate and easily understandable language and sentence structure, even though the message contents were found to have no relation with the main title of the poster. The comprehension of informants could be observed from the knowledge of informants towards the benefit and objectives of the content messages and their efforts on applying them [20]. The conclusion from the results of the interview was that although the informant stated that contents of the conveyed messages were comprehensible, their comprehension was not in accordance with the main purpose discussing stress prevention and the advice to remain optimistic.

#### Acceptability

In this poster media, the acceptability response delivered by the informants showed that the poster did not offend them, and the message content did not contradict the adopted norm. However, there was an opinion that slightly offended an informant who was a smoker. Nevertheless, this was not regarded as an issue because the contents of the message were in accordance with the current conditions. This issue was in line with the study by Arsyanti in 2017 [21], which showed that when media is easily acceptable and understandable, the interest of readers using the media will also increase.

#### Self-Involvement

Informants felt they were involved with the recommendations conveyed via the poster titled “Avoid Stress and Stay Optimistic.” Based on the interview results, there were several informants who provided suggestions on how to deliver messages that were more inclusive, to make the poster more globally applicable and not aimed only at those who were engaged in SFH and WFH. In addition, the issues listed on the poster would likely emphasize more on how to avoid stress and remain optimistic during the pandemic. There were also suggestions to add illustrations, such as those of physical activity and reading books. Illustrations can attract attention and can help to explain and comprehend an issue more easily, clarify important items, and reduce extensive textual descriptions [22].

#### Persuasion

The informants did not have the desire to change their behavior in the near future because they were not interested in the message conveyed through the poster. The provided media was limited, as it focused only on increasing the knowledge and will of people to act, but it was not yet able to influence people to follow the recommendations. The provided health promotion was still limited information, as it has not yet reached the stage of changing the behavior of people [23]. Although mass media campaigns focusing on health promotion have encouraged the target audience to adopt healthy behaviors through messages, they have not been able to achieve the attention required by the media because the messages were perceived to be boring, irrelevant, and difficult to comprehend [24,25]. Moreover, the comprehension of readers could be observed from the knowledge of informants toward the benefit and objectives of the message content and their efforts in implementing those messages [20].

The informants plan to implement the provided advice on avoiding stress and remaining optimistic during the COVID-19 pandemic and consistently continuing to do so. They feel that posters are appropriate reminders, and the target audience is likely to continuously practice it in their daily life. Promotion should not only be limited to providing information, but interesting messages should also be attractive with continuous communication in order to increase motivation among the viewers and to be able to educate the public [23].

### Limitations

When conducting interviews, researchers used a web-based method; therefore, they were unable to observe body movements and facial expressions of the informants. In addition, researchers were unable to control misinterpretation of questions by the informants, which can lead to information bias; therefore, their statements could have different meanings.

### Conclusions

The poster produced by the Ministry of Health, Republic of Indonesia, aimed to serve as an advice to avoid stress and remain optimistic during the COVID-19 pandemic, was still inadequate. Many aspects of the poster needed revisions, such as the visual design, message content, and persuasion, according to the aspects of pretesting communication for effective communication to young adult groups. There was a discrepancy between the title and the recommendations in the content of the poster. In addition, the poster was perceived as unable to persuade the target audience to change their behavior in response to the pandemic. Additional suggestions were made to include illustrations of physical activity, reading books, among others. Hence, pretesting is important to determine how the audience receives the message conveyed through a poster.

## Appendix

Multimedia Appendix 1 Interview result matrix: brief quotes representing answers received by study informants.

## Abbreviations

CDC: Centers of Disease Control and Prevention
CPDI: COVID-19 Peritraumatic Distress Index
IEC: information, education, and communication
SFH: school from home
WFH: work from home
