# Supplementary material for: Pretesting a Poster on Recommended Stress Management During the COVID-19 Pandemic in Indonesia: Qualitative Study
Source: JMIR Form Res. 2021 Sep 23;5(9):e25615. doi: 10.2196/25615 (PMC8462491; doi:10.2196/25615)
Supplement: Multimedia Appendix 1 [file formative_v5i9e25615_app1.docx]

**Multimedia Appendix 1.** Interview result matrix: brief quotes representing answers received by study informants.

**Table S1. Interview result matrix: brief quotes representing answers of informants.**

| **Theme** | **Sub-theme** | **Statements of Informants** |
| --- | --- | --- |
| *Attention* | Title | "We can see that it's clear and boring, if it's from the Ministry of Health it's really ordinary" (P1)  "Very precise, by using persuasive sentences" (P2)  "It’s just ordinary, when I read it, I thought it would discuss about stress, to avoid stress. (P3) "  "The title is suitable, but at first I thought it had nothing to do with Covid" (P5)  "The title is attractive, and looks ……" (P8)  "The title is less attractive" (GD) |
|  | Design | “It’s too plain, it should be fancier, it looks ordinary and unattractive. Add something on the corner….” (P3)  “The design is nice, relaxing…but it’s a little unattractive” (P6)  “Overall, it’s good and attractive” (P7)  “The design of this poster is not good from the size, portion of text and images” (GD) |
|  | Color | “The background color is simple which makes the reader focus on the poster content; the chosen font is just right because it’s easy to read” (P1)  “I think the color is calm but there’s one that’s striking such as the title…” (P4)  “The color is pretty, it’s pleasant to look at” (P7)  “…the color combination is still not right” (GD) |
|  | Font | “The font is suitable. But under the title is too small so I can’t read it” (P3)  “The font of the letters is nice, nothing too much” (P4)  “It’s not unusual, it can be read, but maybe make it bigger because there are lots of empty spaces (P6)  “The size of the letters in several sentences are too small, so it’s hard to read” (P8) |
|  | Lay out | “The writing on the poster is too crowded with so much sentences so when we read it in a glance it’s difficult to understand the conveyed message; to make it more attractive there should be more images added according to the poster messages” (P1)  “I thought these lines had a connection to one another but there wasn’t, I think it’s confusing” (P4)  “It’s already symmetrical, but what is the meaning of those paper plane images? I think it’s just inappropriate to be placed there. The purpose is not clear” (P5)  “The position is quite suitable. But does it mean that these dotted lines have a connection? What does it mean?” (P8)  “The layout of each element is inappropriate and there’s also a meaningless element” (GD) |
|  | Aesthetic Value | “It’s okay, but it’s so ordinary”  “Well, as I said it’s unattractive for the millennials…it’s so ordinary not aesthetic or artsy… (P4)  “I think the color combination is already suitable. It’s aesthetic enough” (P8) |
| *Comprehension* | Message content | “…The title is rather odd, avoid stress and always be optimistic but I think optimistic is ambiguous…The images are also odd because there’s no image on the poster of someone using a cell phone, there’s no advice to read articles on health, communication is made using a phone…” (P3)  “…The title has double messages, the message below it is in small letters, I think just choose one of them” (P4)  “The contents are easy to understand but we have to read it till the end then we can relate with it…at first I didn’t realize it was related to Covid” (P5)  “…the message contents are basic information but I think it lacks research” (GD) |
|  | Sentence structure | “The sentences are okay but it was rather odd because Stress was the title but there wasn’t any explanation on the cause of stress. It just doesn’t relate …” (P3)  I think it’s good because it’s in order, first the title then the points that are in the clouds” (P4)  “The sentence structure is very good and uses the EYD (Enhanced Spelling principles in the Indonesian language) “(P8)  “It’s not to the point, it’s long winded, it should be straight to the point after the title” (GD) |
|  | Language | “Actually, it’s fine but it could be better if it’s persuasive and exciting by using millennial diction” (P4)  “It’s easy to understand. The language is easy, it’s understandable we don’t have to think too hard” (P5)  “The language is simple and easy to comprehend, it’s good” (P8) |
| *Self-Involvement* | Message content with the suitability of the  informant's condition | "To be honest, I think it’s ordinary...okay, I’ll do it and it's appropriate but not exclusively for me" (P3)  "This is what I usually do" (P4)  "It's appropriate, because this condition makes us easily stressed. I think I also seem stressed. The advice is suitable for me, even though it won’t solve the problem either" (P5)  "The message is suitable to my condition. I think overtime it can make us stressful too. So, it’s suitable” (P7) |
| *Acceptability* | Acceptability of  message contents | "There are no offended messages in the message content" (P1)  "Nobody is offended, maybe I'm offended because I’m a smoker, but it's okay, this poster can also remind us to cut down on smoking" (P2)  "Wow, this is what I’ve been doing so, I think it's something usual" (P3)  "I agree with the contents of the message conveyed. Maybe a little offended, but it's okay. Hahaha” (P7) |
|  | Conformity of  material with  norms of the  informant. | "The material is in accordance with the norm" (P1)  "It’s in accordance with the norm but doesn’t quite represent globally because it still lacks images" (P2)  "It’s already according to the norms, nothing violated" (P3)  "In accordance with norms, nothing is diverged" (P4) |
| *Persuasion* | Attractiveness towards persuasion | “In my opinion, persuasion in the poster is quite effective" (P1)  "Actually, I have persuaded others but for me personally it’s not too persuasive so I don't really feel being persuaded" (P3)  "I feel more reminded than persuaded…there are no sentences that really persuades me but it’s enough to remind us all" (P6) |
|  | Impact of message  content | "Very big, it raises awareness of being hygiene during the pandemic" (P2)  "I've actually done what's on the poster, so this poster is just a reminder for me" (P4)  "The message doesn't really have an impact because it's just globally. But the content is good as a reminder" (P6)  "At God’s will the message is acceptable, but I don’t know if it has an impact" (P7) |
|  | Plan of informants  after reading  messages from the poster | "The message content can have an impact to carry out the recommendation" (P1)  "... It's more in our mindset because of the quarantine condition, that’s the activity that we shall do" (P3)  "After reading this poster, I’ll try to be consistent on what I’ve already done ... As a distraction so I don't keep on thinking about Corona" (P5)  "The plan is ... I’ll try to apply everything again. What I haven't implemented is like opening the windows at my house" (P6)  "Yes, I want to try to do it slowly." (P7) |
